# Changes in total volatile organic compound concentration in Seoul subway stations before (2019) and after (2021) the COVID-19 outbreak

**DOI:** 10.1038/s41598-023-46519-9

**Published:** 2023-11-21

**Authors:** Sung Ho Hwang, Jong-Uk Won, Wha Me Park

**Affiliations:** 1 Institute of Health & Environment, Seoul National University, Seoul, South Korea; 2 Institute for Occupational Health, College of Medicine, Yonsei University, Seoul, South Korea; 3 Graduate School of Public Health, Yonsei University, Seoul, South Korea; 4 Department of Occupational and Environmental Medicine, Severance Hospital, Yonsei University Health System, Seoul, South Korea

**Keywords:** Environmental sciences, Risk factors

## Abstract

Volatile organic compounds (VOCs) are major air pollutants often designated as specific hazardous or toxic. This study analyzed the trends in concentration changes and influencing factors of VOCs in underground subway stations in the Seoul Metro before (2019) and after (2021) the COVID-19 pandemic. A total of 506 samples were collected from 253 stations on lines 1–8 between May 2019 and September 2021. Total VOC concentrations in Seoul Metro increased after the COVID-19 pandemic 3.8 times over. The deeper the underground station platform, the greater the difference in the VOC concentrations between 2019 and 2021, which was positively related. Average VOC concentration was the highest (52.8 µg/m^3^) at a depth of 25–30 m and the lowest (23.9 µg/m^3^) at a depth of < 10 m in 2019. In conclusion, excessive disinfection during the COVID-19 pandemic resulted in increased VOC concentrations in the Seoul Metro, especially in the deeper underground stations. Less frequent quarantine disinfection is recommended to improve air quality.

## Introduction

COVID-19 has affected over 252 million people worldwide and has impacted the international economy, industrial production, and social lives of people^1^. Due to the COVID-19 pandemic, many countries have imposed several restrictive measures to prevent the spread of the infection. COVID-19 lockdowns have led to a reduction in gaseous and particulate pollutants in the ambient air^2,3^. Previous studies have also reported the reduced emission of primary indoor pollutants, including volatile organic compounds (VOCs)^4^. These reductions during COVID-19 were a significant improvement in environmental quality parameters with the closed economic activity during lockdowns^3,5^.

**Figure 1:**
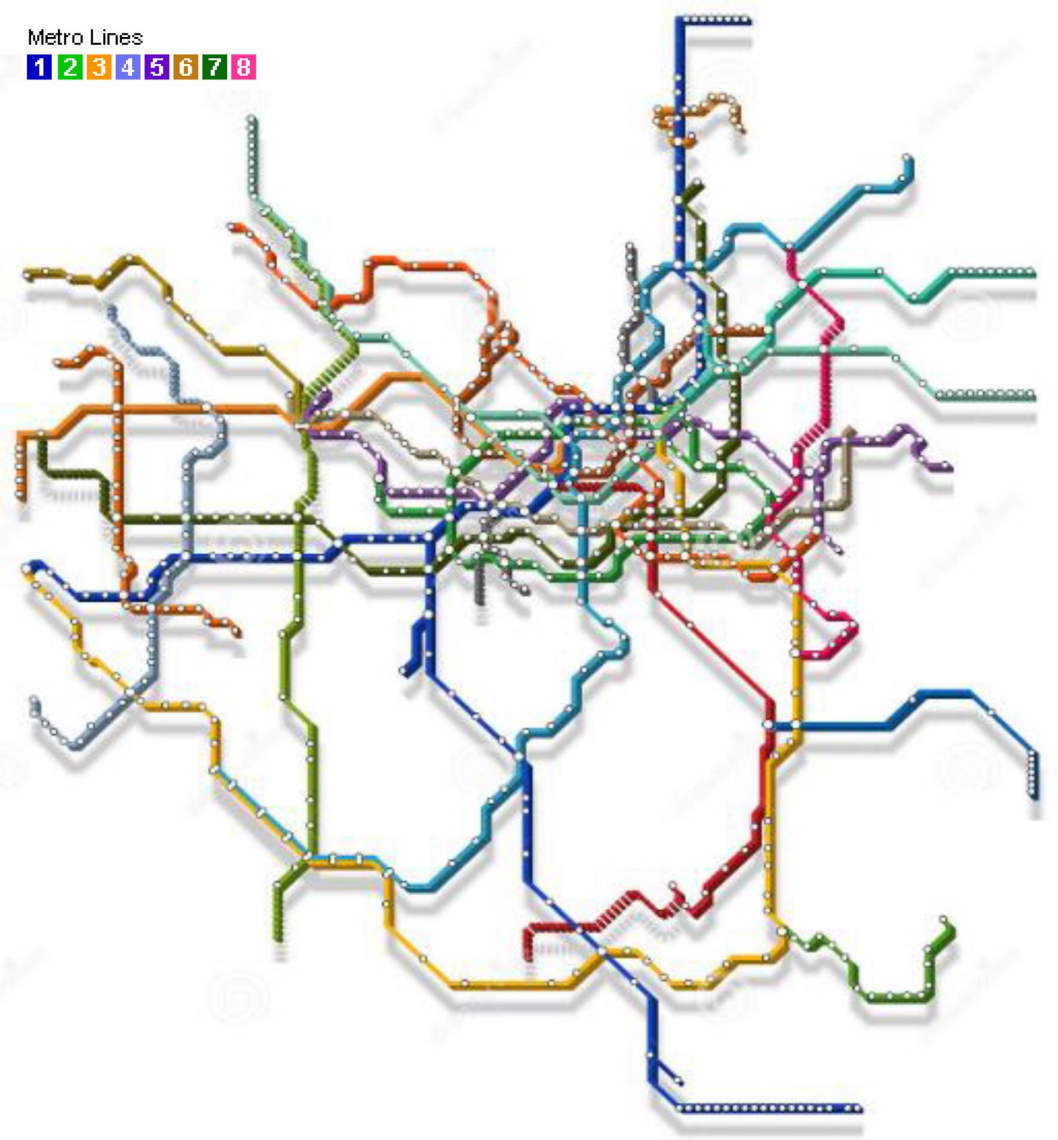
Map of Seoul Metro subway lines 1–9, South Korea.

The Seoul Metro is considered the most used public transportation service in South Korea since 1970 because of its high capacity and reduced traffic congestion^6^. The network consists of numbered lines 1–9 that serve Seoul City proper and its surroundings, as well as other regional railways that serve the greater metropolitan region (Fig. 1). Underground subway stations have confined spaces that can increase the pollutant concentrations entering from the outside atmosphere, in addition to those generated within the system^7^. Previous studies have suggested that air pollutants may influence the severity of COVID-19 associated with respiratory infections^8^. VOCs are considered the main air pollutants and often designated as specific hazardous or toxic air pollutants^9^.

Since COVID-19, Seoul Metro has used more disinfectants during the COVID-19 pandemic than before COVID-19 for sanitation. Disinfectants generally contain chemicals such as alcohol, chlorine, and hydrogen peroxide. These chemicals do not exist in the atmosphere but are released into the atmosphere during disinfection. The disinfectant ingredients are released into the atmosphere, generating VOCs^10^. To protect human health, it is best to limit exposure to products and materials that contain VOCs. Although there are published articles about particulate matter (PM), bioaerosols, and compounds comparing results before and after COVID-19^11–14^, no study on VOCs in underground subway station lines has been conducted to the best of our knowledge.

In the post-COVID-19 era, a better understanding of how factors that affect how future VOC concentrations change depending on the results of policy efforts, such as VOC reduction, will be helpful. In addition, it is necessary to further examine the factors affecting VOC concentrations before and after the COVID-19 outbreak in underground subway stations. In the present study, we investigated the substantial changes in the concentrations of select VOCs before (2019) and after the outbreak of the COVID-19 pandemic (2021) in subway station lines 1–8, respectively.

## Materials and methods

### General information about subway environments

The Seoul Metro is one of the most important commuting infrastructures because of the high number of passengers using this transportation system. General information regarding the Seoul Metro systems in South Korea is shown in Table 1. This includes information on the number of stations and average number of passengers in 2019 and 2021 for subway lines 1–8. After the COVID-19 outbreak, high-performance air purifiers were installed to ventilate the platforms. During the most critical period of the pandemic, the platforms were disinfected at least twice a week, while escalator handrails and elevator buttons, which are in maximum physical contact, were disinfected at least four times a day.

**Table 1:** General information of the subway line system in the Seoul Metro.

Subway line	No. of stations	Construction year	Line length (km)	Average no. of passengers	
				Before COVID-19	After COVID-19
1	10	1974	7.8	17,200,000	11,200,000
2	44	1980	60.2	16,700,000	11,900,000
3	34	1985	38.2	10,100,000	7,070,000
4	26	1985	31.7	13,300,000	8,740,000
5	51	1995	56.9	6,550,000	5,150,000
6	39	2000	36.3	5,530,000	4,070,000
7	51	1996	57.1	7,670,000	5,970,000
8	17	1996	17.7	6,590,000	5,200,000
Total	253				

### Sampling and analysis of VOCs

Sampling was performed at 253 underground stations for 2 years, between May 2019 and September 2021. All 506 samples were collected from the middle area of each underground station platform; 253 samples each were collected in 2019 (between May and September) and 2021 (between May and September). The VOC levels were measured in Tenax-TA tubes using an air sampler (MP-Σ30, SIBATA Scientific Technology Ltd., Japan) at a flow rate of 0.5 L/min for 6 h to obtain VOC sampling.

The analysis of the sample was analyzed by gas chromatograph/mass spectrometry (GC-Clarus SQ 8C GC/MS after desorbing VOCs using turbo matrix thermal desorbers (Perkin Elmer, Shelton, U.S.A) using He (99.999%) and N_2_ (99.999%). The samples were introduced into the instrument by a thermal desorption and sample transfer line 280 °C. Working standard diluted the liquid standard sample, converted it into a gaseous state, and repeatedly injected it in three concentration zones to secure a calibration curve. The total VOC concentration calculation was carried out by converting the chromatogram area into toluene mass units using toluene response coefficients, focusing on general aromatic components, for VOCs detected in the range of n-hexane to n-hexadecane as follows:

$$m_{A} = \frac{{\left( {A_{T} - C_{A} } \right)}}{{b_{st} }}.$$

*m*_*A*_ Amount of analytes in the sample (ng), *A*_*T*_ Sum of analyte peak areas between hexane and hexadecane in the chromatogram of the sample, *b*_*st*_ Calibration curve slope, *C*_*A*_ Vertical axis intercept of calibration curve.

For the quality control of the GC/MS, the correlation coefficient (r_2_) showed good linearity of 0.99 or more as a result of the linearity evaluation, and the instrument detection limit (IDL) experiment showed that the standard deviation (SD) of each material to be investigated was 10% on average, 4% to 17%; the IDL was 0.27 ppbv on average, ranging from 0.18 to 0.35 ppbv; and the relative SD was 2.17.

### Statistical analyses

Statistical analyses were conducted using the R software. A nonparametric analysis was performed because the VOCs were not distributed normally or log-normally according to the Shapiro–Wilk test. Wilcoxon Signed-rank test was performed to determine the differences between overall VOC concentrations in 2019 and 2021 and between VOC concentrations at underground station depths of < 10 m and 25–30 m in 2019 and 2021 to evaluate the effect of the VOC concentration according to the depth of platform in subway stations. Kruskal–Wallis test was used to determine the significance of differences between VOC concentrations at different underground station depths in 2019 and 2021. Spearman’s correlation analyses were used to examine the associations between VOC concentrations and the depth of underground stations as well as the number of passengers at subway stations in 2019 and 2021.

### Consent to participate

All authors give their consent to be co-author in the manuscript.

## Results

VOC concentrations ranged from 1.5 to 566.0 µg/m^3^ with a mean of 43.8 µg/m^3^ in 2019 and 16.7–392.2 µg/m^3^ with a mean of 99.9 µg/m^3^ in 2021 (Table 2). The average VOC concentration in 2021 was 3.8 times higher than in 2019.

**Table 2:** Overall VOC concentration in underground subway stations before and after COVID-19 pandemic.

Line	Length (km)	Before COVID-19 in 2019				After COVID-19 in 2021			
		VOC concentration (µg/m^3^)				VOC concentration (µg/m^3^)			
		Mean ± SD	Min.	Median	Max.	Mean ± SD	Min.	Median	Max.
1	7.8	26.1 ± 19.9	8.5	16.1	60.7	91.7 ± 78.3	19.6	96.6	291.0
2	60.2	32.1 ± 37.7	4.0	20.6	216.0	83.0 ± 52.6	24.2	63.9	218.0
3	38.2	30.7 ± 28.2	1.5	23.2	124.0	64.4 ± 41.8	16.7	54.0	222.0
4	31.7	42.3 ± 61.8	8.3	29.1	291.0	70.6 ± 46.6	21.9	64.4	158.0
5	56.9	54.0 ± 85.7	6.1	30.2	478.0	115.0 ± 71.7	17.1	103.0	392.0
6	36.3	36.3 ± 42.3	7.0	23.9	261.0	124.0 ± 93.9	32.2	99.9	382.0
7	57.1	53.0 ± 82.4	3.6	26.0	415.0	95.1 ± 42.3	36.2	84.3	207.0
8	17.7	65.0 ± 134.0	4.6	19.0	566.0	155.0 ± 83.0	63.5	117.0	360.0
Total	305.9	43.8 ± 69.8	1.5	24.7	566.0	99.9 ± 68.5	16.7	84.4	392.0

**Figure 2:**
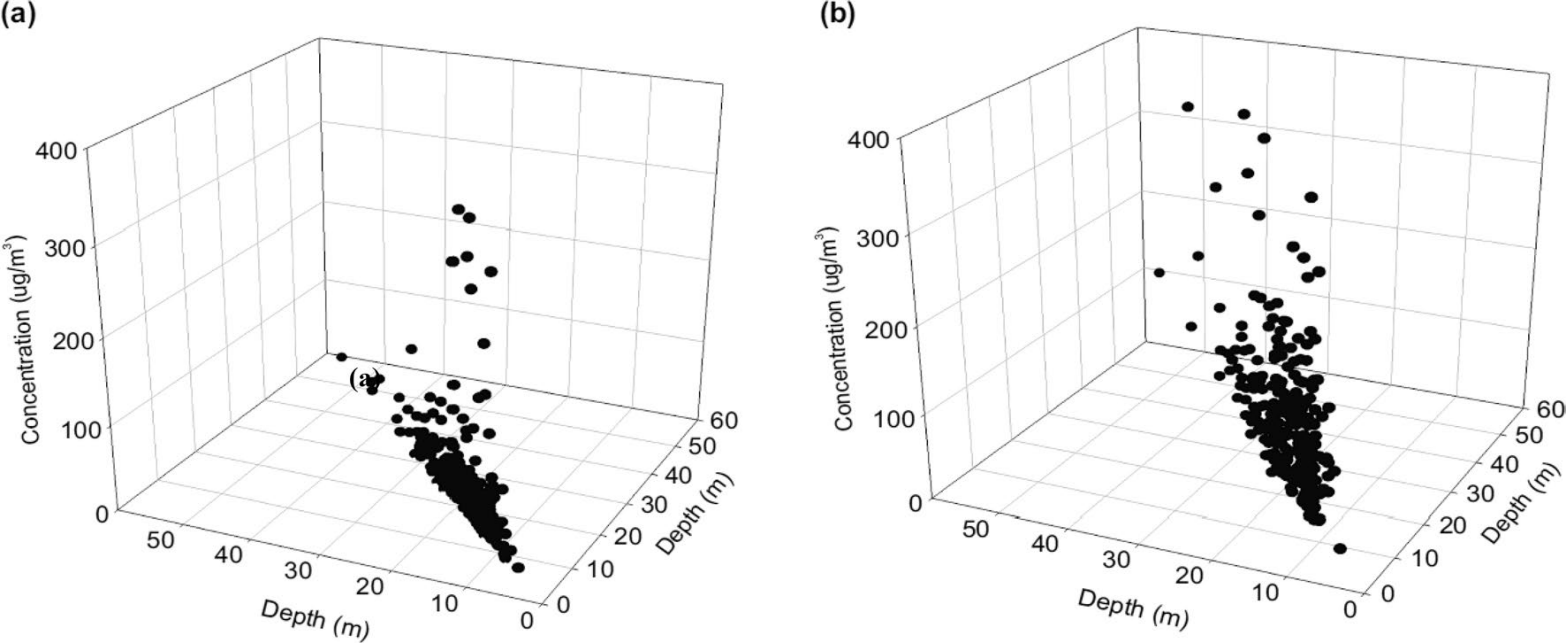
Correlation between VOC concentrations and Seoul Metro station depth in 2019 (**a**) and 2021 (**b**) (p < 0.001, r = 0.17 for (**a**); p < 0.001, r = 0.21 for (**b**)).

**Figure 3:**
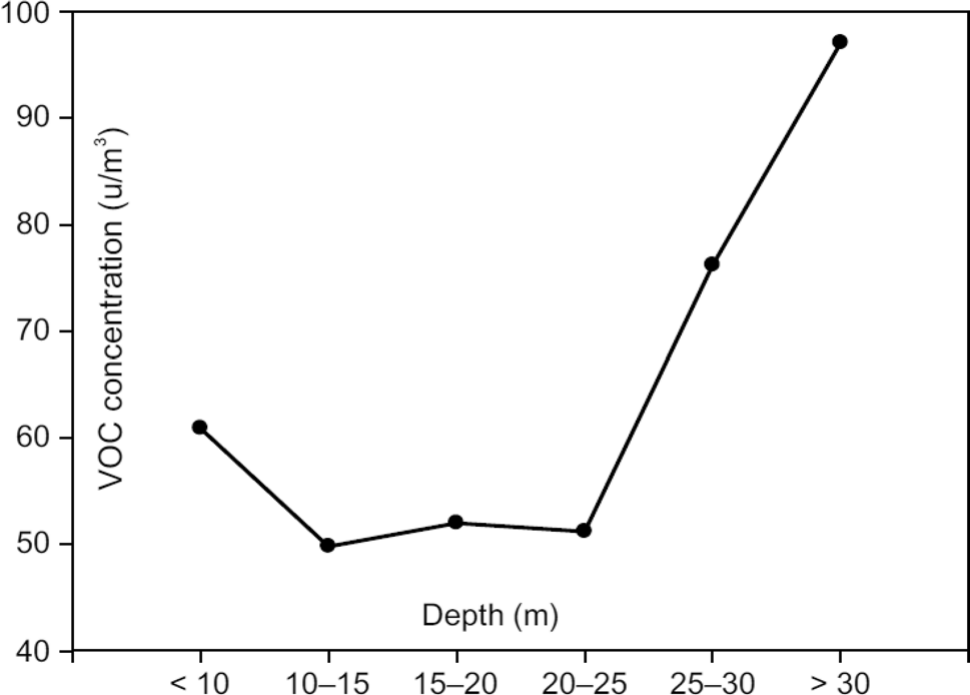
Difference in VOC concentrations between 2019 and 2021 based on depth of underground subway platforms.

Correlation analysis revealed a weak positive association between VOC concentrations and the depth of underground stations in 2019 (p < 0.05, rs = 0.17) as well as 2021 (p < 0.05, rs = 0.21) (Fig. 2). The difference between VOCs concentrations in 2019 and 2021 was monitored between underground depths of < 10 m and > 30 m (Fig. 3). The deeper the underground station platform, the greater the difference between the VOC concentrations in 2019 and 2021.

The VOC concentrations were monitored at underground station depths between < 10 m and > 30 m (Table 3). Average VOC concentration was the highest (52.8 µg/m^3^) at a depth of 25–30 m and the lowest (23.9 µg/m^3^) at a depth of < 10 m in 2019. In 2021, average VOC concentration was the highest (43.8 µg/m^3^) at a depth of > 30 m and the lowest (84.8 µg/m^3^) at a depth of < 10 m. There was a significant difference between the VOC concentrations at different underground depths in 2019 and 2021(p < 0.05) (Table 3).

**Table 3:** VOC concentration in the Seoul Metro based on the depth of underground subway stations before and after the COVID-19 pandemic.

Depth (m)	Before COVID-19 in 2019		Range	p-value^a^	After COVID-19 in 2021		Range	p-value^a^
	VOC Concentration (µg/m^3^)				VOC Concentration (µg/m^3^)			
	Mean ± SD	Median			Mean ± SD	Median		
< 10	23.9 ± 21.8	17.2	4.0–77.4	< 0.05	84.8 ± 36.9	90.6	19.6–160.0	< 0.05
10–15	37.4 ± 70.1	18.7	2.9–566.0		87.2 ± 67.6	66.7	16.7–360.0	
15–20	50.0 ± 63.6	34.3	5.9–332.0		102.0 ± 57.8	104.0	21.2–294.0	
20–25	49.8 ± 77.1	24.9	2.7–415.0		101.0 ± 64.7	84.0	17.1–382.0	
25–30	52.8 ± 95.5	30.9	1.5–478.0		129.0 ± 93.6	103.0	35.3–392.0	
> 30	34.9 ± 30.5	22.8	3.6–128.0		132.0 ± 83.0	101.0	61.6–352.0	

^a^Kruskal–Wallis test.

The average VOC concentration were increased by 56.1 µg/m^3^ when the number of passengers decreased to 2,603,540 in 2021 during the COVID-19 outbreak (Table 4). However, there was no significant correlation between the two variables.

**Table 4:** Comparison between VOC concentrations and number of passengers before and after the COVID-19 pandemic in underground subway stations.

No. of samples	Average no. of passenger	Before COVID-19 in 2019			Average passengers	After COVID-19 in 2021		
		VOC concentration (µg/m^3^)				VOC concentration (µg/m^3^)		
		Min.	Mean ± SD	Max.		Min.	Mean ± SD	Max.
253	9,516,952	1.5	43.8 ± 69.8	566.0	6,913,412	16.7	99.9 ± 68.5	392.0

## Discussion

This study evaluated the variations in VOC concentrations before and after the COVID-19 pandemic in 2019 and 2021, respectively, in the Seoul Metro underground subway station lines 1–8. The total VOC concentrations in 253 underground stations ranged 1.5–566.0 µg/m^3^, with a mean of 43.8 µg/m^3^, in 2019 and 16.7–392.2 µg/m^3^, with a mean of 99.9 µg/m^3^, in 2021. Notably, the mean total VOC concentrations on the underground station platforms for both years were lower than the recommended limit of 400 μg/m^3^^15^. However, the health effects associated with poor indoor environments are most likely driven by chronic low-level exposure to some of these compounds, including gaseous chemicals with a high vapor pressure at room temperature(i.e., VOCs)^16^.

The VOC concentrations in all eight metro lines before the COVID-19 pandemic were lower than those after the pandemic (Table 2). During the pandemic, the average VOC concentration in Seoul Metro underground stations (lines 1–8) increased 2.28 times compared to that before COVID-19. The result was the opposite in underground subway stations with PM, which decreased compared to that before the COVID-19 pandemic^11^. Telecommuting was considered the main reason that PM concentration decreased during the COVID-19 pandemic. This policy considerably reduced the number of passengers using the subway during the outbreak compared to before the outbreak.

The increase in the VOC concentration during the COVID-19 pandemic was attributed to the increased frequency of disinfection. The frequency of the disinfection cycle was increased for all platform structures within the reach of passengers, such as escalator handrails and elevator buttons, to prevent the spread of COVID-19. The Seoul Metro increased the quarantine disinfection frequency to at least twice a week for platforms and at least four times a day for surfaces frequently in physical contact. Frequent quarantine disinfection seems to be a factor that increases VOC concentrations. The use of disinfectants, such as air fresheners and multipurpose surface cleaners, focuses on viral elimination and not necessarily on emissions, with the assumption that more use is better^17^. However, exposure to these cleaning products has been associated with adverse effects on human health. Cleaning products and disinfectants are complex chemical mixtures that often contain multiple respiratory sensitizers and irritants^18^. Nazaroff and Weschler^19^ provided evidence for a link between adverse health outcomes and chemical exposure from cleaning products. Recent nationally-representative population-based studies conducted across the United Kingdom and Sweden found that 32.2% of the general population reported health problems when exposed to all-purpose cleaners and disinfectants^20,21^. Common disinfectants recommended for use against COVID-19 include quaternary ammonium compounds (QAC), hydrogen peroxide, and bleach (sodium hypochlorite). Comprehensive exposure assessment reports for cleaning agents and chemical disinfectants reveal that they include alcohols (ethanol, 928 ± 958 µg/m^3^; and 2-propanol, 47.9 ± 52.2 µg/m^3^); ketones (acetone, 22.6 ± 20.6 µg/m^3^); peroxygen compounds (hydrogen peroxide, < 11.0–511.4 parts per billion (ppb) and peracetic acid, < 2.2–48.0 ppb); monoethanolamines (0.005–0.559 mg/m^3^); ethylene glycol mono-n-butyl ether (49.479 to 58.723 mg/m^3^); benzyl alcohol (0.864–5.446 mg/m^3^); and QAC (benzyldimethyldodecyl ammonium chloride, 0.23 μg/m^3^ and benzyldimethyltetradecyl, ammonium chloride 1.5 μg/m^3^)^22–25^. Frequent disinfection with these substances can affect cleaners who directly handle them; therefore, an overall status and health impact survey of cleaners is needed. Although focusing on different indoor environments, former studies observed that many VOCs are present with disinfectant use during the COVID-19 pandemic^26^.

We confirmed that the depth of the underground platform influences the levels of VOCs, and a greater difference in the levels of VOCs was observed after a depth of 25 m (Fig. 2). Insufficient air exchange rate and improper management of ventilation cause the high VOC levels; therefore, robust indoor air circulation and ventilation enhance the IAQ level and minimize the health risk in subway platforms. The Seoul Metro installed high-performance air purifiers on the platforms to increase the efficiency of ventilation for the underground environment during the COVID-19 pandemic period, thereby providing clean air and a pleasant indoor environment for the platform users. A recent study verified the efficiency of air purifiers in reducing PM concentrations^27^.

The Seoul Metro has been installing highly efficient air purifiers, monitoring IAQ, and frequently cleaning underground stations in an effort to reduce airborne pollution since 2007. However, the VOC concentration was higher during the COVID-19 pandemic than before. The present study found that the deeper the underground station platform, the greater the difference in the VOC concentration between 2019 and 2021 (Fig. 3). This might be attributed to the high frequency of quarantine disinfection, which was conducted at least twice a week for the platform and at least four times a day for escalator handrails and elevator buttons. The Seoul Metro should pay more attention to the ventilation of underground stations for IAQ management and reducing VOC concentration, focusing on platforms at depths n > 25 m, if the use of quarantine disinfectants is to be continued. Alternatively, the frequency of quarantine disinfection using agents that emit VOC pollutants may need to be reduced. A recent study^28^ proposed the use of mitigation strategies, such as air curtains at subway exits, magnetic filters at the top of the ventilation opening, and adsorbing materials that can adsorb harmful gases, to improve the IAQ in underground subway stations. These methods can also be used to reduce the VOC concentrations.

The average VOC concentration increased as the number of passengers decreased during the COVID-19 pandemic (Table 4). Humans are a potent mobile source of VOCs in indoor environments. Several hundred VOCs are emitted into the surrounding air via exhalation and dermal emissions^29^. Oxidants present in indoor air (e.g., ozone or hydroxyl radicals) can produce VOCs by coming in contact with a human body surface (hair and skin) and clothing^30,31^. However, we found there was no significant association between the number of passengers and the VOC concentration, indicating that quarantine disinfection is more likely the main source of VOCs in this study.

Although this is the first study to identify the factors influencing VOC concentrations in Seoul Metro subway stations before and during the COVID-19 pandemic, there are a few limitations. First, the measurements of VOCs in lines 1–8 may not necessarily reflect an association with public health outcomes. Our understanding of the effects of VOCs on human health is limited because of analytical difficulties in measuring real ambient air concentrations and evaluating personal exposure, as well as poor knowledge regarding the toxicity of multiple compounds^32^. Second, other environmental factors, such as ventilation status, air changes per hour, filtering system, temperature, humidity, building materials, and air purifier performance, were not investigated; these factors can affect VOC concentration in underground subway stations. Third, it was impossible to accurately determine the exact air pollutants in the samples because the VOC composition could not be analyzed. Singh^14^ reported that VOCs concentration gradually decreased benzene by − 50% and − 15% for toluene during the lockdown period compared to before the COVID-19 pandemic in 2019, respectively. Then, an increase in the total VOC concentration by 16% was observed in post-pandemic periods; this may be due to the re-opening of commercial places, various industries, and transportation^14^. Finally, the VOC concentration for each station cannot be considered wholly representative because the VOC was measured once for 6 h. Despite these limitations, this study was conducted at 253 underground subway stations with 506 samples in 2019 and 2021 on lines 1–8 at a large scale, which are difficult to access using standard air sampling methods. Our results provide useful indicators for reducing the VOC concentration by reducing the use of disinfectants at depths of over 25 m in underground subway stations. The quantitative concentrations of VOCs are still an efficient indicator for understanding how VOCs can be reduced by applying various systematic methods, such as installing air purification in underground stations, using fewer disinfectants, and increasing the ventilation frequency.

## Conclusion

This study is the first to evaluate the factors affecting the total VOC concentrations in underground subway stations of the South Metro before (2019) and after (2021) the COVID-19 pandemic. The average VOC concentrations in 2021 were 3.8 times higher than in 2019. We found that the deeper the underground station platform, the greater the difference in the VOC concentrations between 2019 and 2021. The increase in total VOCs concentrations during the COVID-19 period is attributed to the excessive quarantine disinfection conducted to prevent the spread of COVID-19 infection. Therefore, the Seoul Metro might need to reduce the frequency of quarantine disinfection, especially in deeper underground stations (> 25 m depth), to improve IAQ. Hence, regular disinfection methods are recommended. Future studies are needed to investigate health effects by comparing human risk assessments according to the exposure of VOCs to individual substances.

## Data Availability

The datasets used or analyzed in this study may be obtained from corresponding authors upon reasonable request.
